# A comparison of the clinical effects of thinning and drilling on laser-assisted hatching

**DOI:** 10.1007/s10103-020-03230-9

**Published:** 2021-01-13

**Authors:** Yujiang Wang, Chuangqi Chen, Jiaying Liang, Lin Fan, Dun Liu, Xiqian Zhang, Fenghua Liu

**Affiliations:** Department of Reproductive Medical Center, Guangdong Women and Children Hospital, No. 521 Xingnan Road, Panyu District, Guangzhou, 511442 Guangdong Province China

**Keywords:** Laser-assisted hatching;, Frozen embryo transfer;, Clinical pregnancy;, Implantation

## Abstract

To systematically investigate the effects of two methods used for laser-assisted hatching (LAH) on clinical outcomes after day 4 (D4) on frozen-embryo-transfer (FET) cycles. Data from 11471 infertile patients who underwent FET cycles between January 2014 and October 2018 was retrospectively analyzed. The 1410 patients who met the inclusion criteria were further categorized into two groups based on the hatching procedure used: the thinning laser-assisted hatching group (T-LAH, 716 patients), and the drilling laser-assisted hatching group (D-LAH, 694 patients). The baseline characteristics of the patients were consistent between the two groups. However, the rates of implantation and clinical pregnancy were significantly higher in the T-LAH group compared to the D-LAH group (32.73% vs. 29.09%, *P* < 0.01, and 50.98% vs. 43.95%, *P* < 0.01). The proportion of live birth was also higher in the T-LAH group, but the difference was insignificant (39.11% vs. 36.89%, *P* > 0.05). Moreover, there were no significant differences in rates of miscarriages, multiple pregnancies, ectopic pregnancies, preterm births, and congenital disabilities between the two groups. Nonetheless, significantly higher rates of implantation and pregnancy were reported in the T-LAH group compared to the D-LAH group among patients aged <35 years, patients with at least one previously failed cycle, and patients with an endometrial thickness of 8–10 mm. T-LAH is superior to D-LAH in improving clinical implantation and pregnancy outcomes in D4 FET, particularly in patients aged <35 years with at least one previously failed cycle or an endometrial thickness of 8–10 mm. The findings of this study provide theoretical support for clinical individualized diagnosis and treatment of patients with infertility.

## Introduction

Mammalian oocytes and early embryos are surrounded by a glycoprotein matrix referred to as the zona pellucida (ZP) [1]. Proteolytic enzymes digest the ZP progressively under specific physiological conditions thus causing it to get thinner as the embryo develops. This goes on until the embryo hatches from ZP to complete implantation [2, 3]. However, in in vitro culture or cryopreservation, a thick or hardened ZP impairs hatching of the human embryo [4, 5]. Various assisted hatching (AH) techniques have been introduced since the advent of mechanical AH to overcome these obstacles [6]. AH can be accomplished through thinning of the ZP using acidified Tyrode’s solution, laser, ZP drilling, two or three-dimensional partial zona dissection using a glass microneedle or a piezo-micromanipulator [7–9]. However, the success of AH is dependent on the method used as well as the extent the AH is done [10].

Laser-assisted hatching (LAH) is a common AH method because of its advantages which include short exposure time, simple operation, accurate positioning, indirect contact, safety, and effectiveness [11]. Zona drilling and zona thinning are the two most frequently used LAH methods in clinical practice. On one hand, zona drilling creates a single full-thickness hole in the ZP that breaks the inner membrane. On the other hand, zona thinning thins a considerable area of the outer ZP layer while keeping the inner layer intact [12]. As such, it poses fewer risks to the embryo compared to zona drilling because there is no hole through which blastomeres can potentially escape or get trapped [13]. Nonetheless, it is still unclear which method provides the best clinical outcomes despite numerous studies comparing the benefits of the two methods. Some studies suggest that T-LAH results in significantly better pregnancy outcomes in human 8-cell embryos compared to D-LAH [14]. Contrary to these findings, Shubhashree et al reported that zona thinning at the 8-cell stage in mouse embryos does not assist nor promote hatching in vitro [15]*.* Most studies compare the clinical effects of assisted incubation and non-assisted incubation, or different assisted incubation methods in the whole infertile population without reporting the populations that benefit from different laser-assisted incubation methods [8]. Herein, the clinical outcomes of T-LAH and D-LAH in day four (D4) frozen-embryo-transfer (FET) cycles were investigated. The effects of the different LAH methods at different embryo ages were also assessed in the context of previously failed cycles and cases of increased endometrial thickness.

## Materials and methods

### Patients’ characteristics

Clinical data from 11471 infertile patients who underwent FET cycles between January 2014 and October 2018 at the department of reproductive medicine of Guangdong Women and Children hospital was analyzed. Patients included in the study were those aged between 22 and 47 years, had undergone Day 4 frozen-thawed embryo transfer, had a maximum of four previous failed IVF-ET procedures, and whose all embryo transfers had been performed using embryos at the morula or cleavage-stage (blastomeres continued to grow after thawing). Finally, only 1410 patients comprising 2915 embryos were included. All the 2915 embryos were frozen on day 3 in vitrification cryopreservation. The 1410 patients were classified into 2 groups based on the laser-assisted hatching method: the thinning laser-assisted hatching group (T-LAH, 716 patients) and the drilling laser-assisted hatching group (D-LAH, 694 patients). This study was approved by the Ethics Committee of Guangdong Women and Children Hospital. All participants provided a written informed consent prior to the study.

### Controlled ovarian hyperstimulation

Patients in both groups underwent controlled ovarian stimulation (COS) using the standard long protocol or the antagonist protocol. The long protocol was administered using GnRHa (1.0–1.2 mg Diphereline) based on the patients’ body mass index during their previous menstruation mid-luteal phase. After 2 weeks, the patients were stimulated using Gn (recombinant human FSH: 100–300 IU). Patients treated using the antagonist protocol were directly treated with Gn from the second to the fifth day of menstruation during the basal follicular phase of ovarian stimulation. Gn doses were adjusted based on ultrasound monitoring and serum E2, LH, and FSH levels. An antagonist (Ghani Rick 0.25 mg) was administered four day post Gn treatment. Patients were treated with human chorionic gonadotropin (hCG; 5,000–10,000 IU) to induce oocyte maturation when a significant number of the follicles reached 16–18 mm. Transvaginal follicular aspiration was then performed 34–36 h later. Regular IVF/ICSI were conducted based on the response of the patients.

### Embryo selection and transfer

Embryos were transferred on day 2 of thawing. Cleavage stage embryos were graded based on the scoring system of the society for assisted reproductive technology (SART). More than 5 cells with a fragmentation rate of less than 25% were cryopreserved. Embryos were thawed and cultured overnight at 37 °C and 6% CO2 before transplantation. Incubation was then done overnight to observe embryo development. Ultrasound scans were also performed to monitor the urinary LH levels of patients with natural ovulation. The freeze-thawed embryos were transplanted on the 3rd day of ovulation (on which the endometrium thickness ≥ 5 mm). Hormone replacement treatment was done in patients without natural ovulation or with irregular menstrual cycles. Estradiol valerate (2–4 mg/day) was prescribed to be taken from the 3rd day of the menstrual cycle. Moreover, the patients were injected with progesterone (80 mg/day) from day 8 to 10. Ultrasound guided embryo transplant was subsequently done on day 11 to 15.

### Freezing and thawing

Freezing and thawing of the embryos was done using previously established protocols [16]. For freezing, the embryos were transferred from the culture media to the basal media (BM) containing HEPES (Quinn’s SAGE, ART-1024) and supplemented with human serum albumin (20% v/v) (HAS, Vitrolife, 10064). The embryos were then transferred to the vitrification solution 1 (BM solution supplemented with DMSO (7.5% v/v) and ethylene glycol (7.5% v/v)) for 2–7 min. They were then transferred to the vitrification solution 2 (BM solution added DMSO (15% v/v), ethylene glycol (15% v/v), and sucrose (10% v/v)) for 30 seconds when shrank to 80%. They were then collected within 5–10 s in a minimal volume and transferred into a cryo device for immediate preservation in liquid nitrogen.

For thawing, the embryos were transferred from the cryo device to warming solution 1 (BM solution added 1 M sucrose) for 1 min and then to warming solution 2 (BM solution added 0.5 M sucrose) for 3 min. They were then transferred to basal medium for 5 min, washed using G2 solution and cultured overnight at 37 °C and 6% CO2.

### Laser-assisted hatching procedure

**Fig. 1:**
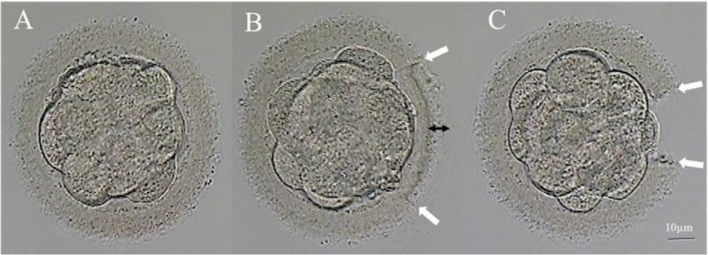
Laser-assisted hatching procedure of vitrified-thawed D4 embryos. **a** D4 morula embryos (non-LAH). **b** T-LAH: ZP was subjected to laser treatment from the outside to the inside until the thickness of the ZP was reduced by 60–80% (represented by the double-ended black arrow) in 25% of the ZP circumference (represented by the double-ended white arrow) **c** D-LAH: ZP was subjected to laser treatment and a 40-μm diameter hole made (represented by the double-ended white arrow)

Laser-assisted hatching was performed using a Zona infrared laser optical system (ZILOS-tk®, Hamilton-Thorne Instruments Biosciences). LYKOS is a unique system in which the laser is built into the objective lens. The system relies on a confocal laser with optical imaging, a 40× objective lens, and 0.6NA. Embryos were exposed to a 1.48-μm wavelength diode laser target positioned to puncture the ZP at the clear space between the inner membrane of the ZP and the blastomere. The positioning ensured that the blastomere was not on the laser path. Few laser pulses at the lowest possible power level were used to limit injury to the blastomeres. The applied laser energy was regulated by controlling the pulse duration of the laser. The pulse width of the laser was set at 25 ns and the typical pulse energy at 300 mJ/pulse. The power of the laser was 100%. Embryos were manipulated in their original culture medium in embryo culture dishes put on the displacement stage of a Diaphot Inverted Microscope. T-LAH was conducted using 5–8 ablations of 2.8 ms successively applied around the ZP. This began at one point and continued until the thickness of the ZP was reduced by 60–80% in 25% of the ZP circumference (Fig. 1b). In the same line, D-LAH was performed using a laser pulse length of 0.450-0.650 ms that made a 40-μm diameter hole in the ZP (Fig. 1c). The culture medium was changed after the procedure. All embryos underwent laser-assisted hatching 2 h before transplantation on the morning of D4.

### Evaluation of clinical outcomes

Clinical pregnancy was determined 4 weeks after embryo transfer through an ultrasound scan. Pregnancy was confirmed if at least one gestational sac was observed, and embryo heart activity was detected. The pregnancy rate was defined as the percentage of ET cycles resulting in pregnancy. The implantation rate was defined as the percentage of gestational sacs of the total number of embryos transferred. The rate of live birth was defined as the proportion of patients with live births among the total transfer cycles. The rate of miscarriage was defined as the number of patients with a miscarriage amongst patients who attained clinical pregnancy.

### Data analysis

All statistical analyses were done using Statistical Product and Service Solutions (SPSS) version 19.0. Data was presented as means ± standard error. *χ*^2^ analysis was used to compare implantation rates between groups. In the same line, the *F* test was used to determine the homogeneity of variance. The independent samples *t* test equal variance while the non-parametric test determined the unequal variance. *P* values less than 0.05 (*P* < 0.05) indicated that there were significant differences between groups.

## Results

### Clinical outcomes of T-LAH vs D-LAH

The basic parameters evaluated using both methods are shown in Table 1. There were no significant differences between the T-LAH and D-LAH group in terms of average age, infertility duration, infertility type, endometrial thickness, the number of transferred embryos, number of previously failed cycles, and embryo survival (*P* > 0.05). Implantation rates and the rates of clinical pregnancies were significantly higher in the T-LAH group(32.73%, 50.98%)compared to those in the D-LAH group (29.09%, 43.95%) (*P* < 0.05 and *P* < 0.01, respectively; Table 2). In the same line, the rate of live births was slightly higher in the T-LAH although the difference was statistically insignificant (*P* > 0.05). In addition, there were no significant differences between the T-LAH and D-LAH group in terms of miscarriages (17.53% vs 13.77%), multiple pregnancies (31.51% vs 34.42%), ectopic pregnancies (3.01% vs 2.62%), preterm births (28.21% vs 32.03%), and birth defects (2.14% vs 1.17%) (*P* > 0.05; Table 2).

**Table 1:** Clinical and laboratory characteristics of the patients in the T-LAH and D-LAH

Characteristic	T-LAH	D-LAH	*P* value
No. of cycles	716	694	
Female age (year, mean ± SD)	33.98 ± 5.31	33.80 ± 5.50	0.266
Duration of Infertility (year, mean ± SD)	4.82 ± 3.67	4.76 ± 3.56	0.378
Infertility diagnosis (%, *n*)			
Female factors	59.78 (428)	56.05 (389)	0.157
Male factors	13.27 (95)	16.43 (114)	0.095
Mixed factors	13.13 (94)	15.13 (105)	0.281
Unexplained infertility	13.83 (99)	12.39 (86)	0.425
Primary/secondary infertility	279/437	291/403	0.257
Treatment of transfer (%, *n*)			
Hormonal replacement cycle	63.27 (453)	64.12 (445)	0.739
Ovulation cycle	6.15 (44)	5.91 (41)	0.851
Natural cycle	30.59 (219)	29.97 (208)	0.801
No. of previous failed cycles	1.41 ± 0.67	1.39 ± 0.70	0.292
Endometrial thickness (mm)	9.21 ± 1.97	9.14 ± 1.85	0.246
No. of embryos thawed	1806	1691	
Embryo survival (%, *n*)	96.01 (1734)	96.10 (1625)	0.899
No. of embryos transferred	1485	1430	
Embryos transferred (*n*, mean ± SD)	2.07 ± 0.44	2.06 ±0 .44	0.335
Morula transferred cycle (%)	71.79 (514)	69.60 (483)	0.366

Values are presented as mean ± standard deviation or number/total (%). No differences were significant (*P* > 0.05)

*T-LAH*, thinning laser-assisted hatching; *D-LAH*, drilling laser-assisted hatching

**Table 2:** Pregnancy outcomes for T-LAH and D-LAH

Outcome	T-LAH	D-LAH	*P* value
No. of cycles	716	694	
Clinical pregnancy (%, *n*)	50.98 (365/716)	43.95 (305/694)	0.008
Implantation (%, *n*)	32.73 (486/1485)	29.09 (416/1430)	0.034
Live birth (%, *n*)	39.11 (280/716)	36.89 (256/694)	0.391
Miscarriage (%, *n*)	17.53 (64/365)	13.77 (42/305)	0.184
Multiple pregnancy (%, *n*)	31.51 (115/365)	34.42 (105/305)	0.423
Ectopic pregnancy (%, *n*)	3.01 (11/365)	2.62 (8/305)	0.762
Birth defects (%, *n*)	2.14 (6/280)	1.17 (3/256)	0.382
Preterm birth (%, *n*)	28.21 (79/280)	32.03 (82/256)	0.336
Low birth weight (%, *n*)	15.56 (54/347)	20.42 (68/333)	0.099

Values are presented as mean ± standard deviation or number/total (%). No differences were significant (*P* > 0.05)

*T-LAH*, thinning laser-assisted hatching; *D-LAH*, drilling laser-assisted hatching

### Comparison of the clinical outcomes across different ages

Comparisons of clinical outcomes among patients of different ages revealed that the rates of implantation and clinical pregnancy were significantly higher in patients aged < 35 years in the T-LAH group (38.35%, 58.04%) compared to those of the same age bracket in the D-LAH group (33.33%, 49.25%) (*P* < 0.05). However, there were no marked significant differences in rates of live birth, miscarriage, and multiple pregnancies (*P* > 0.05) between the groups. Moreover, there were no significant differences in the rates of implantation, clinical pregnancies, live births, miscarriages, and multiple pregnancies in patients aged ≥35 between the two groups (Table 3).

**Table 3:** Pregnancy outcomes related to age

	<35 year			≥35 year		
Outcome	T-LAH	D-LAH	*P* value	T-LAH	D-LAH	*P* value
No. of cycles	398	400		318	294	
Clinical pregnancy (%, n)	58.04 (231/398)	49.25 (197/400)	0.013	42.14 (134/318)	36.73 (108/294)	0.172
Implantation (%, *n*)	38.35 (316/824)	33.33 (271/813)	0.034	25.72 (170/661)	23.50 (145/617)	0.358
Live birth (%, *n*)	45.23 (180/398)	44 (176/400)	0.728	31.45 (100/318)	27.21 (80/294)	0.251
Miscarriage (%, *n*)	14.72 (34/231)	10.15 (20/197)	0.121	22.39 (30/134)	20.37 (22/108)	0.704
Multiple pregnancy (%, *n*)	35.50 (82/231)	37.06 (73/197)	0.738	24.63 (33/134)	27.78 (30/108)	0.579

Values are presented as mean ± standard deviation or number/total (%). No differences were significant (*P* > 0.05)

*T-LAH*, thinning laser-assisted hatching; *D-LAH*, drilling laser-assisted hatching

### Comparison of clinical outcomes in the context of previously failed cycles

Patients were further categorized into two: those receiving the 1st transfer cycle, and those with ≥1 previously failed transfer cycle (Table 4). There were no significant differences between the 2 groups in the rates of implantation, clinical pregnancies, live births, miscarriages, and multiple pregnancies in patients receiving the first transfer cycle (*P* > 0.05). However, in the group with ≥1 previously failed cycles, patients undergoing T-LAH had significantly higher rates of implantation and clinical pregnancies (30.26%, 48.65%) compared with those in the D-LAH group (25.46%, 40.48%; *P* < 0.05). Moreover, there were no significant differences in the rates of live births, miscarriages, and multiple pregnancies in patients with ≥1 previously failed cycle in both groups.

**Table 4:** Pregnancy outcomes related to previous failed cycle

	No previous failed cycle			Previous failed cycle≥1		
Outcome	T-LAH	D-LAH	*P* value	T-LAH	D-LAH	*P* value
No. of cycles	346	363		370	331	
Clinical pregnancy (%, *n*)	53.47 (185/346)	47.11 (171/363)	0.090	48.65 (180/370)	40.48 (134/331)	0.030
Implantation (%, *n*)	35.46 (250/705)	32.56 (238/731)	0.246	30.26 (236/780)	25.46 (178/699)	0.040
Live birth (%, *n*)	40.46 (140/346)	39.94 (145/363)	0.888	37.84 (140/370)	33.53 (111/331)	0.235
Miscarriage (%, *n*)	16.76 (31/185)	14.62 (25/171)	0.580	18.33 (33/180)	12.69 (17/134)	0.176
Multiple pregnancy (%, *n*)	34.59 (64/185)	35.09 (60/171)	0.922	28.33 (51/180)	32.09 (43/134)	0.472

Values are presented as mean ± standard deviation or number/total (%). No differences were significant (*P* > 0.05)

*T-LAH*, thinning laser-assisted hatching; *D-LAH*, drilling laser-assisted hatching

### Comparison of the clinical outcomes in different endometrial thickness

Endometrial thickness was classified as E1 (<8 mm), E2 (8–10 mm), and E3 (> 10 mm) (Table 5). Implantation and clinical pregnancy rates were significantly higher in E2 patients of the T-LAH group (33.01%, 52.46%) than those of E2 patients in the D-LAH group (28.70%, 43.58%) (*P* < 0.05 and *P* < 0.01). However, E2 patients in both groups had insignificant differences in the rates of live births, miscarriages, and multiple pregnancies (*P* > 0.05). E1 and E3 patients in both groups had insignificant differences in all clinical outcomes (*P* > 0.05).

**Table 5:** Pregnancy outcomes related to endometrial thickness

	E<8 (mm)			8-10 (mm)			E>10 (mm)		
Outcome	T-LAH	D-LAH	*P* value	T-LAH	D-LAH	*P* value	T-LAH	D-LAH	*P* value
No. of cycles	118	119		448	436		150	149	
Clinical pregnancy (%, n)	44.07 (52/118)	37.82 (45/119)	0.328	52.46 (235/448)	43.58 (190/436)	0.008	52 (78/150)	46.98 (70/149)	0.385
Implantation (%, n)	28.98 (71/245)	23.14 (56/242)	0.142	33.01 (310/939)	28.70 (258/899)	0.045	34.88 (105/301)	35.29 (102/289)	0.917
Live birth (%, n)	31.36 (37/118)	31.09 (37/119)	0.965	38.39 (172/448)	37.39 (163/436)	0.758	47.33 (71/150)	40.29 (56/139)	0.228
Miscarriage (%, n)	25 (13/52)	15.56 (7/45)	0.252	19.15 (45/235)	13.16 (25/190)	0.098	7.69 (6/78)	14.28 (10/70)	0.197
Multiple pregnancy (%, n)	34.62 (18/52)	24.44 (11/45)	0.275	30.21 (71/235)	35.26 (67/190)	0.269	33.33 (26/78)	35.71 (25/70)	0.761

Values are presented as mean ± standard deviation or number/total (%). No differences were significant (*P* > 0.05)

*T-LAH*, thinning laser-assisted hatching; *D-LAH*, drilling laser-assisted hatch

## Discussion

Vitrification procedures cause hardening of the zona pellucida (ZP) thereby leading to hatching difficulties [17]. Zona thickness is negatively associated with embryo implantation [18]. Assisted hatching improves implantation rates during assisted reproduction [19]. Laser-assisted hatching through zona drilling or thinning significantly increases implantation and clinical pregnancy rates [17, 20]. However, data regarding the approach that provides better clinical outcomes is lacking. Whether the two methods can be used interchangeably in Day4, FET remains unclear. Herein, clinical outcomes in D4 frozen-thawed embryos treated using T-LAH and D-LAH were analyzed. T-LAH treatment resulted in higher rates and significantly higher clinical pregnancy rates compared with D-LAH treatment. Moreover, T-LAH treatment resulted in slightly higher live birth rates compared with D-LAH treatment. However, the two approaches had no significant differences in the rates of miscarriages, multiple pregnancies, ectopic pregnancies, preterm births, and congenital disabilities. These findings suggested that patients who underwent T-LAH had better clinical outcomes.

The clinical effects of T-LAH and D-LAH at various ages, frozen embryo transfer cycles, and endometrial thicknesses were also further analyzed. The physiological embryo hatching process can be explained using two theories [21]. The first theory postulates that the digestive enzymes that degrade the ZP mediate embryo hatching [22]. The second theory postulates that embryo hatching results from physical pressure from the proliferating blastomere and the associated expansion and contraction of the blastocoel cavity [23]. ZP thickness varies in infertile women. Moreover, the in vitro culture environment influences the thickness of ZP. Long co-incubation periods generate elevated levels of reactive oxygen species which damage the embryos by hardening the ZP. This in turn affects embryo development [24]. In addition, embryo freezing and thawing, female age, embryo quality, and culture medium affect the texture of ZP and prevent embryo hatching and implantation [25]. Successful implantation requires synchronous development of both the embryo and the endometrium. These happenings cause the embryo to express and secret multiple factors that promote its attachment to the endometrium via the ZP thereby improving the clinical outcomes [26]. As such, a systematic clinical analysis is required to determine which method(s) has optimal clinical outcomes.

The efficiency of ZP thinning decreases significantly with advancing age thereby reducing the implantation rates [27]. LAH is conducted on frozen embryos before FET to reverse the adverse effects of zona hardening and improve the chances of successful pregnancy [28]. Nonetheless, some studies report that assisted hatching does not improve reproductive outcomes in advanced maternal age [29]. These contradictory results are attributed to the use of different sample sizes or different assisted hatching methods [30]. The Chinese Society of Reproductive Medicine (CSRM) data suggest that more than 70% of patients receiving assisted reproductive technology (ART) treatment in China are aged below 35 years [31]. In the same line, the US CDC report indicates that majority of patients receiving infertility treatment are aged below 35 years old (38.3%) and between 35 and 37 years (21.5%) [32]. These reports indicate that the rate of infertility among the young people in China is higher than that of USA. It is widely known that age is an independent risk factor for female fertility and pregnancy outcome. The ovarian function usually has a significant downward trend from the age of 35 [33]. The Chinese practice guideline on ART strategies for women with advanced age use 35 years as the dividing line for female infertility patients; older patients are generally defined as those aged above 35 years [34]. Herein, the rates of implantation and clinical pregnancy in patients aged below 35 years were significantly higher in the T-LAH group (38.4%, 58.0%) compared to those of the D-LAH group (33.3%, 49.3%) (*P* < 0.05) . Similar trends were observed in patients aged 35 years and older though the differences were insignificant.

Hardening of the ZP and embryo hatching difficulties causes poor pregnancy outcomes in patients with multiple implantation failures even in the absence of other additional factors [35]. These implantation failures result from poor embryo-endometrial synchronous FET [36]. AH is beneficial to patients with a history of implantation failures. The patients undergo fresh cleavage-stage embryo transfer [37]. Successful implantation needs synchronous development of the embryo and the endometrium. AH prolong the interaction time between embryo and endometrium thereby improving embryo attachment to the endometrium. LAH through ZP thinning significantly enhances the clinical outcomes by improving the rates of implantation and clinical pregnancies FET among patients with recurrent failure [38]. Herein, patients with previously failed implantation of D4 frozen-thawed cycles exhibited higher implantation and clinical pregnancy rates with T-LAH treatment compared to D-LAH treatment. This implied that T-LAH confers beneficial clinical outcomes for D4 vitrified-warmed embryos. However, there were no significant differences between the outcomes of the two approaches for patients undergoing first embryo transfer cycles. Analysis of the clinical outcomes in the context of varied endometrial thicknesses further revealed that patients with an endometrial thickness of 8–10 mm had significant improvements in implantation and clinical pregnancy rates under T-LAH treatment compared to D-LAH treatment. This strongly suggested that T-LAH treatment is superior to D-LAH treatment.

Similar studies using mouse models report that zona thinning at the 8-cell stage does not aid nor promote hatching in vitro [15]. However, it is unclear whether human embryos behave in a similar manner under similar conditions. Honguntikar, S. D. et al reported that laser manipulation (zona opening) significantly enabled blastocyst hatching. However, DNA damage in laser-hatched embryos affected implantation and post-implantation development [39]. Given that the ZP inner membrane is broken to designate a full-thickness opening, the embryos can suffer sudden change in the biochemical environment with limited time for adaptive changes, are at a higher risk of contracting a bacterial infection or immunologic aggression, and the blastomere can be easily lost through the hole or separation/splitting thereby causing monozygotic twinning [20]. Herein, clinical merits and demerits of applying either of the two methods were critically analyzed. Evidently, T-LAH significantly improved the clinical outcomes of the patients compared to D-LAH. This was notably observed in implantation and clinical pregnancy rates in D4 FET cycles among patients aged <35 years with previous implantation failures (≥1 cycles) as well as those with an excellent endometrial thickness (8–10 mm). This phenomenon was hypothesized to have been influenced by two factors. DNA damage to the laser-hatched embryos negatively affected their implantation and post-implantation development despite the zona opening having better hatchability and survival rate than zona thinning. Moreover, thinning caused the embryos to express and secrete multiple factors which promoted their attachment to the endometrium. Nonetheless, further studies should be conducted to test these hypotheses.

In the same line, the rate of live births was slightly higher in the T-LAH group compared to the D-LAH group (39.11% vs. 36.89%). However, the difference was insignificant. Similarly, there were no significant differences between the T-LAH and D-LAH group in terms of adverse effects during pregnancy and newborns. This finding further affirmed that thinning is a safe and feasible approach for assisted hatching.

Nevertheless, this study was limited by lack of embryo hatching rates of the two groups. Cognizant to this, comprehensive prospective studies should be conducted in the future to comprehensively explore the clinical outcomes of the two LAH methods.

## Conclusion

This study systematically analyzed the clinical effects of two assisted incubation methods in populations of different ages. T-LAH was found to be superior to D-LAH in improving clinical implantation and pregnancy outcomes in D4 FET, particularly in patients aged less than 35 years with at least one previously failed cycle or an endometrial thickness of 8–10 mm. The findings of this study provide theoretical support for clinical individualized diagnosis and treatment of patients with infertility.

## References

[1] Ferré M, Amati-Bonneau P, Morinière C, Ferré-L'Hôtellier V, Lemerle S, Przyrowski D, Procaccio V, Descamps P, Reynier P, May-Panloup P (2014). Are zona pellucida genes involved in recurrent oocyte lysis observed during in vitro fertilization?. J Assist Reprod Genet.

[2] Cohen J (1991). Assisted hatching of human embryos. J Assist Reprod Genet.

[3] Balakier H, Sojecki A, Motamedi G, Bashar S, Mandel R, Librach C (2012). Is the zona pellucida thickness of human embryos influenced by women's age and hormonal levels?. Fertil Steril.

[4] Sun ST, Choi JR, Son JB, Joo JK, Ko GR, Lee KS (2012). The effect of long zona dissection using ICSI pipettes for mechanical assisted hatching in vitrified-thawed blastocyst transfers. J Assist Reprod Genet.

[5] Kirienko KV, Apryshko VP, Naumova AA, Yakovenko SA (2019). Mechanical zona pellucida removal of vitrified-warmed human blastocysts does not affect the clinical outcome. Reprod BioMed Online.

[6] Cohen J, Elsner C, Kort H, Malter H, Massey J, Mayer MP, Wiemer K (1990). Impairment of the hatching process following IVF in the human and improvement of implantation by assisting hatching using micromanipulation. Hum Reprod.

[7] Balaban B, Urman B, Alatas C, Mercan R, Mumcu A, Isiklar A (2002). A comparison of four different techniques of assisted hatching. Hum Reprod.

[8] Alteri A, Viganò P, Maizar AA (2018). Revisiting embryo assisted hatching approaches: a systematic review of the current protocols. J Assist Reprod Genet.

[9] Nakayama T, Fujiwara H, Yamada S, Tastumi K, Honda T, Fujii S (1999). Clinical application of a new assisted hatching method using a piezo-micromanipulator for morphologically low-quality embryos in poor-prognosis infertile patients. Fertil Steril.

[10] Mantoudis E, Podsiadly BT, Gorgy A, Venkat G, Craft IL (2001). A comparison between quarter, partial and total laser assisted hatching in selected infertility patients. Hum Reprod.

[11] Le MT, Nguyen T, Nguyen T, Nguyen VT, Le DD, Nguyen V, Cao NT, Aints A, Salumets A (2018). Thinning and drilling laser-assisted hatching in thawed embryo transfer: a randomized controlled trial. Clin Exp Reprod Med.

[12] Chunyan Y, Haiying Z, Mahmoud M, Ahmed A, Jiaxiang H, Ashraf E-S, Jianghua S, Qingyou L (2019) Effects of laser zona thinning and artificial blastocoel collapse on the cryosurviving and hatching of buffalo (Bubalus bulalis) blastocysts of different ages. Theriogenology. 10.1016/j.theriogenology.2019.11.015 10.1016/j.theriogenology.2019.11.015 31767184

[13] Liu C, Su K, Shang W et al (2020) Higher implantation and live birth rates with laser zona pellucida breaching than thinning in single frozen-thawed blastocyst transfer. Lasers Med Sci. 10.1007/s10103-019-02946-7 10.1007/s10103-019-02946-7 31897814

[14] Jeong JE, Joo BS, Kim CW, Kim HG, Joo JK, Lee KS (2018). Effects of three-area laser-assisted zona thinning in 8-cell human embryos on pregnancy outcomes in vitro fertilization. Clin Exp Reprod Med.

[15] Uppangala S, D’Souza F, Pudakalakatti S, Atreya HS, Raval K, Kalthur G, Adiga SK (2016). Laser assisted zona hatching does not lead to immediate impairment in human embryo quality and metabolism. Syst Biol Reprod Med.

[16] Wang Y-j, Liu W-j, Fan L, Li Z-t, Huang Y-q, Chen C-q, Liu D, Zhang X-q, Liu F-h (2020). The impacts of the number of prefreeze and postthaw blastomeres on embryo implantation potential: A systematic analysis. Medicine.

[17] Zeng M, Su S, Li L (2018). Comparison of pregnancy outcomes after vitrification at the cleavage and blastocyst stage: a meta-analysis. J Assist Reprod Genet.

[18] Edi-Osagie E, Hooper L, Seif MW (2003). The impact of assisted hatching on live birth rates and outcomes of assisted conception: a systematic review. Hum Reprod.

[19] Martins WP, Rocha IA, Ferriani RA, Nastri CO (2011). Assisted hatching of human embryos: a systematic review and meta-analysis of randomized controlled trials. Hum Reprod Update.

[20] Ali J, Rahbar S, Burjaq H, Sultan AM, Al Flamerzi M, Shahata MA (2003). Routine laser assisted hatching results in significantly increased clincal pregnancies. J Assist Reprod Genet.

[21] Practice Committee of the American Society for Reproductive Medicine, & Practice Committee of the Society for Assisted Reproductive Technology (2014). Role of assisted hatching in in vitro fertilization: a guideline. Fertil Steril.

[22] Park SB, Kim HJ, Choi YB, Ahn KH, Lee KH, Yang JB, Yu CS, Seo BB (2014). The effect of various assisted hatching techniques on the mouse early embryo development. Clin Exp Reprod Med.

[23] Dunstan GR (1990). Human embryos: the debate on assisted reproduction. J Med Ethics.

[24] Liu J, Zhang X, Yang Y, Zhao J, Hao D, Zhang J, Liu Y, Wu W, Wang X (2016). Long-time vs. short-time insemination of sibling eggs. Exp Ther Med.

[25] Hammadeh ME, Fischer-Hammadeh C, Ali KR (2011). Assisted hatching in assisted reproduction: a state of the art. J Assist Reprod Genet.

[26] Greco E, Litwicka K, Arrivi C, Varricchio MT, Caragia A, Greco A, Minasi MG, Fiorentino F (2016). The endometrial preparation for frozen-thawed euploid blastocyst transfer: a prospective randomized trial comparing clinical results from natural modified cycle and exogenous hormone stimulation with GnRH agonist. J Assist Reprod Genet.

[27] Kanyo K, Zeke J, Kriston R, Szücs Z, Cseh S, Somoskoi B, Konc J (2016). The impact of laser-assisted hatching on the outcome of frozen human embryo transfer cycles. Zygote.

[28] McLaughlin JE, Choi BY, Liu Q (2019). Does assisted hatching affect live birth in fresh, first cycle in vitro fertilization in good and poor prognosis patients?. J Assist Reprod Genet.

[29] Tannus S, Cohen Y, Henderson S, Son W-Y, Tulandi T (2019). The effect of assisted hatching on live birth rate following fresh embryo transfer in advanced maternal age. Reprod Sci.

[30] Kissin DM, Kawwass JF, Monsour M, Boulet SL, Session DR, Jamieson DJ (2014). Assisted hatching: trends and pregnancy outcomes, United States, 2000–2010. Fertil Steril.

[31] Yang J, Deng C, huang X (2020). Chinese Society of Reproductive Medicine (CSRM) Chinese Society of Reproductive Medicine Annual Report: data analysis of ART in 2017. J Reprod Med.

[32] National Centers for Chronic Disease Prevention and Health Promotion (2016) Assisted Reproductive Technology National Summary Report (USA) [EB/OL] 2018-10. Available at: https://www.cdc.gov/art/reports/2016/national-summary.html. Accessed 28 Dec 2018.

[33] National Collaborating Centre for Women’s and Children’s Health (UK) (2013). Fertility: assessment and treatment for people with fertility problems. London R Coll Obstetr Gynaecol.

[34] Jiang L, Chen Y, Luo X (2019). Chinese Society of Reproductive Medicine (CSRM). Chinese practice guideline on the assisted reproductive technology (ART) strategies for women with advanced age. Chin J Evid Based Med.

[35] Nakagawa K, Takahashi C, Nishi Y, Jyuen H, Sugiyama R, Kuribayashi Y, Sugiyama R (2012). Hyaluronan-enriched transfer medium improves outcome in patients with multiple embryo transfer failures. J Assist Reprod Genet.

[36] Lee J, Cha J, Shin S (2019). Effects of laser-assisted thinning versus opening on clinical outcomes according to maternal age in patients with repeated implantation failure. Lasers Med Sci.

[37] Friedler S, Schachter M, Strassburger D, Esther K, Ron El R, Raziel A (2007). A randomized clinical trial comparing recombinant hyaluronan/recombinant albumin versus human tubal fluid for cleavage stage embryo transfer in patients with multiple IVF-embryo transfer failure. Hum Reprod.

[38] Lu X, Liu Y, Cao X (2019). Laser-assisted hatching and clinical outcomes in frozen-thawed cleavage-embryo transfers of patients with previous repeated failure. Lasers Med Sci.

[39] Honguntikar SD, Uppangala S, Salian SR, Kalthur G, Kumar P, Adiga SK (2015). Laser-assisted hatching of cleavage-stage embryos impairs developmental potential and increases dna damage in blastocysts. Lasers Med Sci.

